# Transient windows for connectivity in a changing world

**DOI:** 10.1186/2051-3933-2-1

**Published:** 2014-01-13

**Authors:** Sara L Zeigler, William F Fagan

**Affiliations:** Department of Biological Sciences, Virginia Tech, Blacksburg, VA 24060 USA; Department of Biology, University of Maryland, College Park, MD 20742 USA

**Keywords:** Anthropogenic change, Dispersal, Disturbance, Dynamic landscapes, Functional connectivity, Movement, Static landscapes, Structural connectivity

## Abstract

The primary focus of studies examining metapopulation processes in dynamic or disturbance-dependent landscapes has been related to spatiotemporal changes in the habitat patches themselves. However, like the habitat patches, opportunities for movement between patches can also exist intermittently in dynamic landscapes, creating transient connectivity windows – which we define as a period of time during which matrix conditions increase the probability of one or more individuals moving successfully between habitat patches. Far less is known about the implications of dynamic changes in connectivity *per se*, and, to our knowledge, there are no connectivity metrics or metapopulation models that explicitly consider intermittent changes to connectivity between habitat patches. Consequently, in this paper, we examined the peer-reviewed, published literature up to November 2013 to better understand the consequences of variability in connectivity and to highlight knowledge gaps on this topic. First, we describe how connectivity *per se* can vary along a temporal gradient, offering examples of ecological systems that fall along this gradient. Second, we examine how temporal variability in connectivity is important for metapopulation dynamics, particularly given likely alterations to disturbance regimes as a result of global change. We conclude our review by briefly discussing key avenues for future connectivity-related research, all of which hinge on the need to perceive connectivity as a transient feature.

## Introduction

From an organism’s perspective, landscapes can vary in two ways: (1) spatially, through patchiness in habitat suitability, and (2) temporally, through variability in the extent, distribution, and quality of habitat over time [1]. As a result, landscapes can span a complex spectrum from highly ‘static’, with little spatiotemporal habitat variability, to highly ‘dynamic’, with near-constant turnover in habitat amount, quality, and/or configuration through time. Examples of largely static environments include contiguous expanses of long-lived forests that do not experience large-scale disturbances (e.g., redwood forests, *Sequoia sempervirens*), whereas dynamic environments include, for example, disturbance-dependent successional forests, agricultural landscapes, and ephemeral pool complexes.

Many researchers, particularly those writing in the disturbance ecology literature, have characterized the features of landscapes and their implications along this static-dynamic continuum. For example, because habitat is often disturbance-driven in dynamic landscapes, patch quality (as viewed from the perspective of early colonizers) declines over successional time. Such declining quality can result in local extinctions for early successional species, which can be offset by novel opportunities for recolonization that can occur as new habitat patches are created elsewhere [2, 3]. Thus, metapopulations in dynamic environments tend to have higher extinction risk [2, 4] and lower occupancy levels [4–10] compared to those in static environments because habitat destruction (or degradation) increases local extinction rates [4–9] and decreases the amount of habitat available for colonization at any one point in time [6]. Extinction risk in dynamic landscapes is further exacerbated by so-called refractory periods in which patches are not suitable for successful colonization immediately following their creation [11]. Similarly, autocorrelation in disturbance timing can augment extinction risk in dynamic landscapes by reducing the amount of habitat available at any one time [12, 13]. In addition, key elements of life history, population dynamics, and habitat needs are predicted to differ widely for species along the spectrum of static to dynamic landscapes [4, 6, 14, 15]. Therefore, processes that cause turnover in habitat quality, amount, and/or configuration through time have major impacts on metapopulation dynamics.

Despite this long-standing recognition of the unique characteristics of dynamic landscapes, the literature has emphasized the effects of spatiotemporal changes to the habitat patches themselves, while paying less attention to dynamism in connectivity. However, just as the habitat patches themselves are ephemeral, opportunities for movement between patches or populations can also exist intermittently in dynamic landscapes [4, 15], creating transient connectivity windows. We define a transient connectivity window as a period of time during which conditions increase the probability of one or more individuals moving successfully between habitat patches.

Intermittent movement opportunities arise through some temporary biotic or abiotic change to the matrix separating populations on suitable habitat patches [16]. Such changes to the connectivity of the matrix may or may not involve the creation of new suitable habitat. For example, clearcutting in Finnish forests converts unsuitable, late-successional matrix to suitable early successional habitat for the marsh fritillary butterfly (*Euphydryas aurinia*), promoting connectivity between more stable meadow habitats by making the matrix more structurally similar to habitat patches [16, 17]. In contrast, marine ecosystems provide numerous examples in which transient connectivity is of great importance for demographic processes but is unrelated to structural connectivity. For example, off the coast of California, phenological shifts in the primary direction of near-shore surface currents determine the directionality of larval transport, creating opposing patterns of source-sink dynamics in two congeneric mussel species [18]. Similarly, in the eastern Atlantic Ocean, currents retain most hatching loggerhead sea turtles in the immediate vicinity of their nesting grounds in the Cape Verde Islands, but, on occasion, the currents shift and send hatchlings southeastward to the coast of Sierra Leone (R. Scott, pers. comm.). Other examples of transient connectivity windows that are unrelated to physical structure include cases where species occupancy patterns determine functional connectivity. For instance, certain avian species are more likely to cross forest boundaries into open matrix habitat in the presence of tufted titmice (*Baeolophus bicolor*), leading to movement between patches in risky landscapes [19]. In all of these examples, movement opportunities are short-lived relative to the timescales of the patches themselves, leading to transient windows of connectivity. As we detail later in the paper, the frequency and duration over which such connectivity windows remain open varies across ecological systems.

Even though habitat connectivity is often critical to the persistence of individual populations, metapopulations, and species [20, 21], spatiotemporal changes in habitat connectivity *per se* have received far less attention from ecologists compared to changes in habitat patch availability or quality. Particularly lacking is a synthetic treatment of the full spectrum of transient connectivity and how such connectivity affects ecological dynamics. For example, much of what is known about metapopulation extinction risk and occupancy is derived largely from models that assume constant pathways of connectivity among habitat patches [22–24] (but see [6]). Consequently, in this paper, we examined the peer-reviewed, published literature up to November 2013 to better understand the consequences of variability in connectivity and to highlight knowledge gaps on this topic. First, we describe how connectivity *per se* can vary along a temporal gradient, offering examples of ecological systems that fall along this gradient. Second, we examine how temporal variability in connectivity is important for metapopulation dynamics, particularly given likely alterations to disturbance regimes as a result of global change. We conclude our review by briefly discussing key avenues for future connectivity-related research, all of which hinge on the need to perceive connectivity as a transient feature.

## Review

### Transient connectivity windows along a temporal gradient

**Figure 1:**
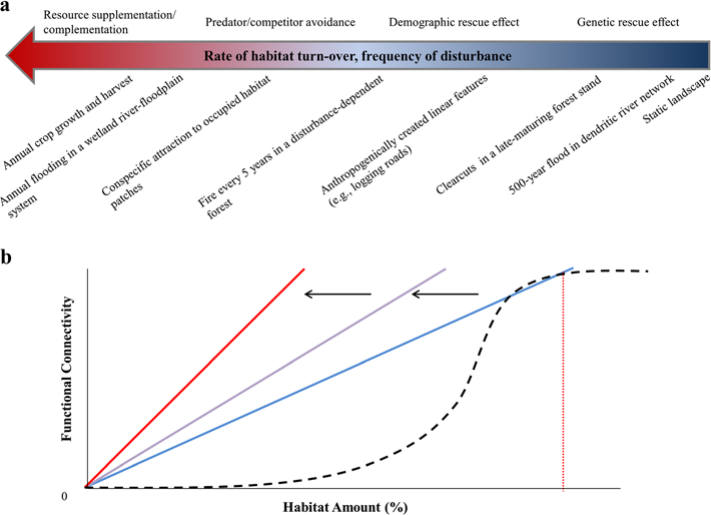
**Connectivity along a temporal gradient. (a)** Windows of connectivity in systems (examples below arrow) fall along a temporal gradient controlled by the frequency of change in matrix conditions. This gradient ranges from systems where windows of connectivity are short and infrequent (right, blue region of arrow) to systems where windows of connectivity open seasonally or very frequently (left, red region of the arrow). As connectivity increases, the immigration rate is also predicted to increase, leading to heightened effects of connectivity as one moves along the temporal gradient (text above arrow). **(b)** We predict that connectivity and its benefits to population persistence should increase as one moves along the temporal gradient from static landscapes (dashed line) to increasingly dynamic landscapes (solid lines with colors representing placement of landscape on gradient). In addition, static landscapes have a threshold amount of habitat (red dashed line) at which point minimal decreases in habitat amount cause a rapid decrease in connectivity (With et al. 1997). However, dynamic habitats tend to show a linear relationship between habitat amount, and connectivity lacks a similar threshold (Hanski [6]).

In dynamic environments, levels of connectivity can differ for a given species as a direct result of changes in abiotic or biotic matrix conditions. Habitat patches that appear isolated at a given snapshot in time may at other times be structurally or functionally connected as newly created, more suitable matrix conditions provide temporary opportunities for movement, creating “bridges” between subpopulations that appear and disappear through time. Thus, dynamic landscapes tend to have higher connectivity with the same amount of habitat as static landscapes [4, 15], and connectivity increases with increasing rates of turnover in matrix conditions [4] (Figure 1). In addition, connectivity typically declines nonlinearly with decreasing habitat quantity in static landscapes (i.e., where matrix conditions generally do not change through time) such that a threshold exists where the landscape becomes rapidly disconnected with only a small amount of additional habitat loss [25]. In contrast, given the more complex transient nature of connectivity in dynamic landscapes, this threshold effect may not exist, causing connectivity to decline linearly with habitat loss [6] (Figure 1).

Dynamic landscapes also exhibit a high degree of temporal variability in the turnover of suitable matrix conditions, which we summarize by placing landscapes along an associated temporal gradient (Figure 1). This gradient reflects the frequency with which movement opportunities appear between any two habitat patches and is dependent on a given species’ generation time or life history attributes. Along this gradient, movement opportunities can range from very rare events that occur only once every few generations (low landscape connectivity) to very frequent, seasonal events that can occur multiple times within a single generation (high landscape connectivity; Figure 1). Where an ecological system or landscape falls along this gradient can have important implications for evolutionary processes and metapopulation dynamics, which we will describe in more detail in the next section.

#### Landscapes where rare disturbances create fleeting movement opportunities

**Figure 2:**
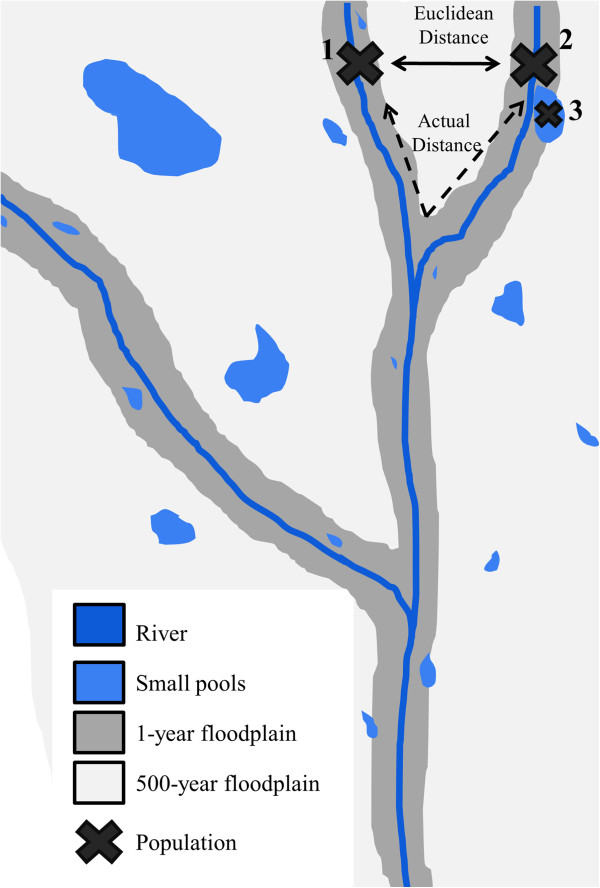
**Transient windows of connectivity in a dendritic river system.** In this example, the out-of-network Euclidean distance separating two populations of a fish species (black X’s) in the river is short, while the actual in-network dispersal distance is much longer, preventing connectivity between the two populations. A third population exists in a small pool in the river’s floodplain and is completely isolated from populations in the river. Temporal windows of connectivity at varying frequencies, however, allow these populations to be connected through time. An annual flood (dark gray) would connect populations 2 and 3 each year, while a much rarer 500-year flood (light gray) would connect all populations. The frequencies of these transient windows of connectivity could have important impacts on metapopulation persistence, gene flow, and habitat occupancy for the species.

In some landscapes, dynamic changes in matrix conditions are rare and take place over long time scales (e.g., decades or centuries), occurring once every several generations for species inhabiting these landscapes. Consider a dendritic, aquatic ecosystem, where terrestrial matrix is completely unsuitable for the movement of strictly aquatic species. Due to the branching geometry of riverine habitats, populations separated by only short Euclidean distances may actually be remote for species limited to in-network movement [26] (Figure 2). However, years with unusually high precipitation or rare regional flooding events could offer sporadic hydrological links between unconnected populations within the river network, creating short-lived opportunities for movement. Infrequent events that facilitate connectivity, through either within- or out-of-network dispersal, can be important for the maintenance of genetic diversity, population persistence, and metapopulation stability, especially in the upper reaches of dendritic systems [26, 27].

Similarly, forestry practices can create relatively infrequent connectivity windows in terrestrial systems [28]. For example, timber harvest in a stand of a late maturing, long-lived trees may open late successional matrix vegetation and create movement opportunities between forest gaps for early successional species [17]. Movement opportunities may exist until matrix suitability declines with succession, at which point the transient window of connectivity may remain closed for decades until the stand is ready for a subsequent harvest.

#### Landscapes where more frequent disturbances create movement opportunities within a single generation

In other landscapes, the mechanisms that alter matrix conditions occur over much shorter, more frequent time intervals, creating movement opportunities one or more times within a generation. For example, many species depend on movement opportunities that occur following relatively frequent, low intensity wildfires that create pathways among otherwise disconnected habitats. Fire promotes connective pathways for red-cockaded woodpeckers (*Picoides borealis*) in the southeastern United States as individuals preferentially disperse through areas with low densities of hardwood trees and young pines [29]--conditions maintained by wildfires that historically occurred every 3–5 years [30]. Likewise, recently burned habitat (i.e., every 9 years) is critical for the movement of Florida scrub lizards (*Sceloporus woodi*), a low-vagility species that inhabits early successional scrub habitats [31].

In addition, marine currents can vary episodically, creating pathways for larval dispersal [18]. Many marine species disperse only during the larval stage, and dispersal pathways are determined by local fluid mechanical processes influenced by, for example, wind direction and water temperatures [32]. In many systems, a unidirectional flow field primarily moves larvae away from their natal sites. As a result, recruitment levels fall below replacement levels, causing population declines [32]. Episodic current reversals that occur, for example, during El Niño events are therefore critical for maintaining stable metapopulations and preventing local extinctions [32].

#### Landscapes where seasonal disturbances or highly frequent events create short-lived connectivity cycles

In still other systems, successful movement requires changes to matrix conditions on highly frequent or seasonal time scales that occur many times within a single generation. For instance, weather can alter matrix conditions daily in a way that opens and closes transient connectivity windows; four butterfly species in the Netherlands had an increased dispersal propensity and rate of colonization on sunny versus cloudy days [33].

Several examples from freshwater systems make clear the importance of seasonal cycles in connectivity. For instance, annual precipitation in the Florida Everglades is concentrated from May to October, causing water levels to rise and providing temporary hydrological links between permanent water bodies during the rainy season [34]. Small fish species use these transient connectivity windows to actively spread throughout the wetland ecosystem, where they are able to escape predation pressure, grow, and reproduce [35, 36]. As water levels recede during the dry season, hydrological connectivity changes, eliminating temporary overland links and concentrating fish in permanent water bodies [34–36]. Similar patterns have been observed in the Mulligan River, an ephemeral river in the Australian arid zone [37]. In general, movement opportunities created by flooding between rivers and small, isolated water bodies throughout the surrounding floodplain allow fish access to complementary habitats necessary for certain parts of their life cycle and to refugia from competitors and large, less-vagile piscivores during the breeding season [38]. Seasonal connectivity also allows for the exchange of nutrients, organic matter, zooplankton, and invertebrates between the floodplain and water channel [38, 39]. Through these mechanisms, transient windows of connectivity between river networks and their floodplains ultimately result in a higher diversity of fish and amphibians [38], and species survival and persistence often depend on the interplay between a species’ dispersal ability and the seasonal increase in habitat connectivity [40].

#### Environments where movement opportunities are highly variable

Our final category pertains more to “environments” (as opposed to physical landscapes) with transient connectivity windows, in which we include any system where movement opportunities are impacted by the presence or actions of other organisms and are thus highly variable over time. To illustrate, certain boreal mosses, such as *Tetraplodon angustatus*, use the droppings of large mammals as habitat patches and rely on flies for spore dispersal [41]. In this system, the matrix between dung piles is unsuitable for between-patch movement by the mosses, unless the presence of spore-carrying flies “improves” matrix conditions. Thus, short-term variations in the presence of these flies allows for transient windows of connectivity between populations on separate dung piles.

In another example, some species preferentially immigrate to habitat patches already occupied by their own species or by ecologically similar heterospecifics. Here, patch-level connectivity patterns are intimately related to local occupancy patterns, which are themselves dynamic on short time scales [42–45]. If the presence of a conspecific or heterospecific causes a disperser to move toward a habitat patch, occupied patches could draw dispersers more often than would be otherwise expected. This would result in movement to occupied habitat patches where connectivity might not otherwise be expected, increasing the overall level of functional landscape connectivity [46]. For example, red-cockaded woodpeckers preferentially disperse to habitat patches that already contain breeding individuals [29]. Similarly, some species are more likely to move between habitat patches when a second “support species”, such as tufted titmice, are present in the matrix itself [19]. Again, functional connectivity is enhanced by the presence of a particular species. The opposite pattern would be expected if the presence of a conspecific (e.g., for species displaying territorial behaviors) or another species (e.g., a predator) causes a disperser to intentionally avoid moving into the matrix or dispersing to an occupied habitat patch. In either case, the occupancy status of a habitat patch or the matrix, which is itself a dynamic trait, alters how a disperser views matrix conditions and controls the duration of the connectivity window in the landscape.

In these examples, the frequency and duration of the connectivity windows are highly variable, depending on the dynamics of other individuals or species. For species that preferentially move to occupied patches or that move among patches in the presence of other species, we would expect windows of connectivity that open frequently and remain open for long periods when the population is abundant and highly dispersed throughout the landscape (i.e., because there is a high probability that individuals are present in other patches or in the matrix at large population sizes). However, we hypothesize that the reverse would be true for species at low population sizes or with patchy distributions. Here, the probability that the matrix or another patch is occupied would be low, reducing the frequency and duration of connectivity windows. A connectivity window would thus only open very rarely given a chance dispersal event that causes an empty patch to become occupied. Additional research on such systems is needed to better understand temporal variability in connectivity when connectivity is related to the dynamics of other species or individuals.

### Impacts of transient connectivity windows

The spatial structure and connectivity patterns (whether static or dynamic) within metapopulations can govern epidemiological, genetic, demographic, and other population-level processes. In some ecological systems, subpopulations benefit from the movement of individuals between habitat patches and thrive in landscapes with high levels of connectivity. High levels of landscape connectivity or frequent opportunities for movement might allow an individual to forage across multiple habitats [47], to supplement or complement its available resource base [48], or to access refugia during vulnerable time periods (e.g., the breeding season; [35, 38]). At the population level, high landscape connectivity can allow for more rapid recolonization of extirpated patches [49] and promote demographic or genetic rescue effects for declining populations through immigration [50, 51]. In such ecological systems, the persistence of both occupied patches and metapopulations is correlated with high levels of habitat connectivity [20, 21], and extinction rates tend to decrease with increasing immigration rates by reducing inbreeding and dampening stochastic fluctuations [22]. In other ecological systems, connectivity between populations can have negative consequences, allowing for the spread of parasites and diseases [52] or promoting outbreeding depression in highly adapted subpopulations [53]. In these situations, metapopulation and subpopulation extinction risk tend to decline with increasing immigration rates.

In dynamic environments, immigration rate is controlled by the frequency and duration of transient connectivity windows (i.e., by the rate of change in matrix conditions), and, as a result, the benefits or drawbacks of connectivity should also fall along the temporal gradient of connectivity (Figure 1). For instance, only species inhabiting dynamic landscapes with fast-paced changes in matrix conditions (i.e., species inhabiting landscapes on the left/red section of the connectivity gradient in Figure 1) should be able to move often enough within their lifetimes to benefit from access to a broader resource base [47, 48] or to refugia [35, 38].

However, ecological systems can still be affected by transient changes in connectivity even when those connectivity windows only open over multi-generational or geological time scales. Studies suggest that populations can benefit from genetic rescue, where the introduction of new alleles from immigrants reduces inbreeding depression and genetic drift [51], with as few as one immigrant per generation or longer [54]. In this case, subpopulations may benefit from genetic rescue even in landscapes where transient connectivity windows open rarely or only for short periods of time (i.e., landscapes to the right/blue section of the connectivity gradient in Figure 1). Furthermore, Benzie [55] and Palumbi et al. [56] noted that patterns of genetic differentiation in populations of a variety of marine species, including starfish (*Acanthaster* spp.), giant clams (*Tridacna* spp.), and urchins (*Echinometra* spp.), do not match contemporary ocean currents. These authors suggest that episodic changes to the physical environment over geological time scales, such as changes in sea level, have periodically removed dispersal barriers and allowed rare, intermittent pulses of dispersal that have shaped the genetic structures of these organisms. Similarly, very rare, episodic “taxon pulses” that occurred as dispersal barriers were temporarily removed may have allowed the evolution of complex host-parasite relationships [57]. However, whether a burst of immigrants arriving in one pulse has the same genetic or evolutionary effects as a slow but steady dribble of colonists depends very much on the genetic makeup of the arrivals and the state of the recipient population.

Over intermediate time-scales (i.e., occurring one or more times within a generation), we would expect that a moderate number of immigrants (i.e., a moderate frequency of movement opportunities) would be necessary for a population to benefit from the demographic rescue effect [50], where immigrants can compensate for low survival or recruitment within a population [54]. In contrast, dynamic environments on the right of the gradient (i.e., where rare disturbances create fleeting movement opportunities) should be the least vulnerable to widespread disease outbreaks and more likely to build a genetic structure conducive to local adaptation.

Furthermore, the sum-total impacts of connectivity in dynamic environments are likely to be quite complex. On one hand, as discussed above, overall connectivity within the entire metapopulation is predicted to be higher in dynamic landscapes than in static landscapes. Thus, a single gene or a disease is more likely to spread throughout the metapopulation, explaining, for example, the weak genetic structure and lower levels of population differentiation for Mallee emu-wren (*Stipiturus mallee*) inhabiting fire-dependent woodlands in Australia [58]. On the other hand, connectivity between a given pair of sub-populations is predicted to be lower in a dynamic landscape, where movement opportunities are not constant compared to “connected” subpopulations in a static landscape. Thus, subpopulations may not experience all of the effects of connectivity, depending on the rate of change in matrix conditions. As a result, the balance between positive and negative aspects of landscape connectivity will hinge on the timescales over which such connectivity occurs.

Ultimately, however, little is actually known regarding the full impacts of transient windows of connectivity or of changes to the frequency and duration of those windows in dynamic landscapes. Additional research that evaluates the temporal aspects of connectivity in dynamic environments is needed. This should include research in empirical systems as well as the development of connectivity metrics and metapopulation models that can incorporate spatiotemporal variability in connectivity.

### Implications of anthropogenic change

Because species in dynamic landscapes may have specific windows of time in which to move between habitat patches, anthropogenic alterations to the system (e.g., climate change, landcover change) could have disproportionately large impacts on such species. Climate change, for instance, has already had major impacts on Earth’s physical systems [59, 60], increasing air and water temperatures, pushing local precipitation regimes towards their extremes, and amplifying the frequency of extreme weather events [59]. In addition, a number of studies have documented changes to disturbance regimes, including increases in the number of large fires, floods, and hurricanes [61].

Climate-related changes in connectivity will have context-specific consequences. In some ecological systems, such changes will likely have negative consequences for endemic species. For example, polar bears (*Ursus maritimus*) rely on winter ice thickening for movement, which opens seasonal connectivity windows between habitat patches and populations. However, warming temperatures attributed to climate change have drastically shortened the ice season in some places, leading to habitat fragmentation [62] and reduced mating opportunities, survival, and reproduction [62, 63]. In other systems, climate change may have positive impacts on species in dynamic environments. For example, higher temperatures and drier conditions have contributed to an increased extent and frequency of wildfires in Catalonia, Spain, which has increased connectivity and persistence for common linnets (*Carduelis cannabina*) and woodlarks (*Lullula arborea*) [64]. In addition, increased rainfall or variability in rainfall related to climate change is predicted to improve connectivity and reduce genetic differentiation for hairy footed gerbils (*Gerbillurus paeba*) in a semiarid savanna [65]. In such cases, increased disturbance frequencies due to climate change may actually increase movement opportunities, enhancing the benefits of demographic and genetic rescue effects.

In addition to climate change, anthropogenic landcover change can also have major impacts to the temporal dynamics of connectivity. Dynamic aquatic systems appear to be particularly vulnerable to this type of change. Rivers are routinely dammed, diverted, channelized, and pumped, which dramatically alters natural flooding regimes, reduces windows of connectivity in the system, and negatively impacts resident species [39]. For example, the channelization and damming of the Danube River in the mid-1800’s altered the river ecosystem, which was once characterized by flooding-related dynamic changes in connectivity, into a static landscape with limited connectivity. This change has led to significant terrestrialization and a decline in species richness [66]. Similarly, the increase in impervious surfaces that accompanies urbanization can lead to more frequent, rapid changes in water levels and a lower frequency of flooded or saturated conditions for nearby vernal pools, reducing windows of connectivity between pools and negatively impacting resident species [67]. Finally, anthropogenic changes to disturbance regimes can alter windows of connectivity and impact population-level processes, particularly genetic ones [68]. Fire suppression, for instance, has reduced connectivity for collared lizards (*Crotaphytus collaris*) in the Missouri Ozarks, changing how genetic differentiation is partitioned within and between subpopulations [68].

In contrast, anthropogenic change can increase connectivity levels in other ecological systems. For example, humans create unpaved secondary roads (e.g., for logging), railways, verges along roads and railways, and power lines that are maintained through regular disturbance and land clearing. Wolves (*Canis lupis*) as well as several butterfly and grass species opportunistically use of these linear formations as movement corridors that connect populations [69–72]. However, windows of connectivity close when these linear features are not maintained through, for example, mowing. In these examples, increased connectivity through anthropogenically created corridors has had a positive effect in promoting (meta)population persistence [69–72]. However, we could envision examples where the development of these temporary corridors could link populations and allow for the spread of disease or promote movements that increase predation along well-traveled pathways. Additional research is needed to understand the implications of human-induced connectivity patterns.

Finally, anthropogenically driven extinctions and population declines could have impacts on associated species that have previously not be considered. For species that are more likely to disperse to occupied habitat patches, we hypothesize that a “connectivity Allee effect” could be possible, where movement between populations is drastically reduced at very low population and occupancy levels. In addition, for species that are more likely to move through matrix in the presence of another species or that require another species for movement, we would also hypothesize that the loss of the “support species” from the system could result in the loss of movement opportunities. Given the pervasiveness of species declines and extinctions throughout the world, connectivity reductions through the loss of interdependent species are likely commonplace, and future research on this concept is needed.

In general, anthropogenic change in dynamic landscapes can lead to subtle, but important, shifts in the frequency and duration of transient connectivity windows. In many cases (although not all), these windows will likely open less frequently and for shorter periods as lands are developed, water systems are altered, ecological communities are disrupted, and temperature, precipitation, and disturbance regimes shift [62, 66, 68]. As a result, connectivity will decline in these systems, which may reduce colonization rates, decrease levels of demographic and genetic rescue, alter genetic structure, and ultimately increase extinction risk. Reduced connectivity will have particularly strong consequences for species that will need to track climate change in order to persist [73].

However, to our knowledge, broad discussions of anthropogenic changes rarely, if ever, include how such changes may lead to altered connectivity regimes. This lack of attention is especially disconcerting given the wide variety of disturbance-dependent, dynamic landscapes throughout the world (as exemplified in this review). Instead, recent studies on the impacts of anthropogenic change have typically focused on factors other than connectivity (e.g., phenology, demographic rates, habitat availability, and geographic distribution; [74–76]). However, all of these features and others (e.g., population declines, range shifts, species extinctions, and altered community structures) often hinge on connectivity. Consequently, research focusing directly on the impacts of anthropogenic change on connectivity in dynamic environments will be critical to adequately understand and manage vulnerable metapopulations on a changing planet.

## Conclusions

In dynamic landscapes, opportunities for movement exist during limited windows of connectivity as matrix conditions change through time. As a result, the frequency and duration of those transient connectivity windows can have major implications for metapopulation processes. In addition, anthropogenic influences (acting through a variety of mechanisms, such as temperature changes, alterations to precipitation and disturbance regimes, and landcover change) may shift the frequency with which transient connectivity windows open, resulting in a host of population-level consequences (e.g., inbreeding depression, population decline, species extinction) in some dynamic environments. However, despite the fact that connectivity is arguably more important in dynamic environments [77], few studies have directly measured or focused on dynamism in connectivity *per se*.

In an important and recent comprehensive review of the state of connectivity research, Kool et al. [78] marginally addresses connectivity in dynamic landscapes, noting instead the rarity of information on this topic. These authors make clear that analyzing connectivity in dynamic landscapes is difficult, as spatiotemporal changes on the landscape can confuse observations [78]. At a minimum, researchers evaluating connectivity in dynamic landscapes (e.g., through circuit or graph theoretic approaches) should assess likely changes in connectivity over long time periods to adequately understand connections between populations [32]. This point is further supported in a review by Williams and Hastings [32], who indicated that the use of time-averaged connectivity patterns is insufficient for determining persistence in metapopulations existing in temporally varying networks and will often result in an over-estimation of the probability of persistence. A structured approach that incorporates time-varying measures of connectivity is needed.

Furthermore, metapopulation models have routinely either assumed (1) a constant colonization and extinction rate that is not influenced by the number of immigrants (e.g., the Levins model [23]) or (2) that connectivity between subpopulations is constant, even if colonization and extinction rates are impacted by the number of immigrants [22]. Hanski [6] created a more appropriate model for metapopulations in dynamic landscapes. However, even this model does not address the possibility that connectivity could change through time, considering only that the number of possible connections to a given patch changes as other patches appear and disappear. Each of these models has been used to predict thresholds for metapopulation persistence or to characterize equilibrial occupancy levels – information that could be important for understanding and managing the dynamics of declining species. Thus, a metapopulation model that considers temporally changing opportunities for connectivity through time could be especially useful for understanding metapopulation persistence in disturbance-dependent ecological systems.

In summary, we highlight the following aspects of transient connectivity that require additional research:

- Development of approaches (i.e., metrics, tools, etc.) for analyzing and measuring landscape connectivity through time in dynamic landscapes.
- An explicit consideration of the impacts of anthropogenic change (through climate change, landcover change, and alterations to community structure) on patterns of connectivity *per se* and of the implications of such changes in empirical systems.
- Development of metapopulation models that explicitly consider dynamic changes in connectivity among subpopulations.
- A better understanding of how transient connectivity affects population-level processes, such as gene flow, disease spread, and metapopulation persistence.
- A better understanding of how habitat loss interacts with transient connectivity to affect system-level fragmentation, including the potential for threshold-type collapses.

Filling these knowledge gaps is a critical step towards a more complete understanding of landscape connectivity on a changing planet.

## Authors’ information

SZ is currently a postdoctoral research associate in the Department of Biological Sciences at Virginia Tech with Dr. Jeffrey Walters where her research centers on the development of models that simulate how landcover change and management in longleaf pine ecosystems impact population persistence of red-cockaded woodpeckers. Her work generally focuses on the intersection of landscape processes and population dynamics for endangered species conservation.

WF is a Professor and Chair of the Department of Biology at the University of Maryland. He has broad-ranging interests in spatial and theoretical ecology, including questions related to animal migration and nomadism, phenology, succession, and extinction risk.
